# Post SARS-CoV-2 vaccination Guillain-Barre syndrome in 19 patients

**DOI:** 10.6061/clinics/2021/e3286

**Published:** 2021-09-28

**Authors:** Josef Finsterer, Fulvio A. Scorza, Carla A. Scorza

**Affiliations:** I Klinik Landstrasse, Messerli Institute, Vienna, Austria.; II Disciplina de Neurociencia, Escola Paulista de Medicina/Universidade Federal de Sao Paulo (EPM/UNIFESP), Sao Paulo, SP, BR.

**Keywords:** SARS-CoV-2, COVID-19, Neuro-COVID, Complications, Vaccination, Polyradiculitis

## Abstract

SARS-CoV-2 vaccinations are not free from side effects. Usually, they are mild or moderate but occasionally severe. One of these severe side effects is Guillain-Barré syndrome (GBS). This review summarizes and discusses GBS as a side effect of SARS-CoV-2 vaccinations (SCoVaG) based on recent research reports. Altogether, nine articles reporting 18 patients with SCoVaG were identified and one more report on another patient is under review. The age for the studies ranged between 20-86y. Nine patients were male, and ten were female. In all 19 patients, SCoVaG developed after the first dose of the vaccine. The Astra Zeneca vaccine was used in fourteen patients, the Pfizer vaccine in four patients, and the Johnson & Johnson vaccine was applied in one patient. The latency between vaccination and onset of GBS ranged from 3h to 39d. The treatment of SCoVaG included IVIGs (n=13), steroids (n=3), or no therapy (n=3). Six patients required mechanical ventilation. Only a single patient recovered completely and partial recovery was achieved in nine patients. In conclusion, GBS may develop time-linked to the first dose of a SARS-CoV-2 vaccination. Though a causal relationship between SARS-CoV-2 vaccinations and SCoVaG remains speculative, more evidence is in favour than against it.

## INTRODUCTION

Guillain-Barré syndrome (GBS) is increasingly recognized as a complication of infections with SARS-CoV-2 (COVID-19) (1). At the end of December 2020, at least 220 patients with SARS-CoV-2 associated GBS were reported (1). Between January 2021 and the end of June 2021, at least 75 more patients with this complication were reported. Whether the number of patients with SARS-CoV-2 associated GBS has been declined since the introduction of SARS-CoV-2 vaccines is unknown. Nevertheless, SARS-CoV-2 vaccines are not free from side effects and may not only cause mild or moderate adverse reactions but even severe complications, including neurological side effects (2). Though generally rare, these neurological side effects are increasingly recognized and reported, including SARS-CoV-2 vaccination associated GBS (SCoVaG). This review summarises and discusses the pathophysiology, clinical presentation, treatment, and outcome of SCoVaG.

## METHODS

A literature search was conducted using the databases PubMed and Google Scholar and applying the search terms “SARS-CoV-2 vaccination”, “mRNA based vaccine”, “vector-based vaccine” combined with “side effect“, “adverse reaction”, “polyradiculitis”, “neuropathy”, “Miller-Fisher syndrome”, “polyneuritis cranialis”, “Bickerstaff encephalitis” and “Guillain-Barre syndrome”. Additionally, reference lists from the available articles were further checked. Articles that provided detailed information about individual patients experiencing any subtype of GBS after the first or second dose of the SARS-CoV-2 vaccine were included. At the same time, articles that were not accessible or only available as an abstract or in a language other than German, English, French, or Spanish were excluded.

## RESULTS

Altogether nine articles reporting 18 patients with SCoVaG were noted (3-11). A study on one more patient has not been published yet. The age of the 19 patients ranged between 20 and 86y (Table 1). Nine patients were male, and ten were female (Table 1). SCoVaG developed in all the patients after the first dose of the vaccine (Table 1). The Astra Zeneca (ASZ) vaccine was used in 14 patients, the Pfizer vaccine in 4 patients, and the Johnson & Johnson vaccine was used in 1 patient. The latency between vaccination and onset of GBS ranged from 3h to 39d (Table 1). The treatment of SCoVaG included IVIGs (n=13), steroids (n=3), or no therapy (n=3) (Table 1). Additionally, two patients required plasmapheresis as IVIGs were ineffective. One patient received only pregabalin because of dysesthesias, and six required artificial ventilation (Table 1). In two patients, therapeutic management has not been reported. Only one patient was reported to achieve complete recovery, partial recovery was achieved in nine patients, and the outcome was not reported in nine patients (Table 1).

All four patients reported from the study of Allen et al. (3) experienced bifacial muscle weakness with paresthesias within three weeks after administering vector-based SARS-CoV-2 vaccine being classified as polyneuritis cranialis (PNC) variant of GBS (3). Three patients benefitted from intravenous immunoglobulins (IVIGs) or steroids, and one recovered without treatment (3). None of the four patients required mechanical ventilation.

The fifth patient with SCoVaG was a 32y old male who developed GBS, subtype acute, inflammatory, demyelinating polyneuropathy (AIDP), eight days after the first dose of a vector-based SARS-CoV-2 vaccine (Table 1) (4). This patient had a previous history of AIDP 14 years earlier, from which he recovered completely and benefitted from two IVIG cycles and plasmapheresis but is still handicapped and currently undergoing immune-adsorption (4).

The sixth patient with SCoVaG was a 69y old female who developed AIDP 39d after the first dose of a vector-based SARS-CoV-2 vaccine and after 15d was tested positive for SARS-CoV-2 (Table 1). The case has not been published yet but is under review.

The seventh patient with SCoVaG is an 86y old female diagnosed with GBS one day after receiving the first dose of an mRNA-based SARS-CoV-2 vaccine (Table 1) (5). She recovered only partially under IVIGs (5) and did not require mechanical ventilation.

The eighth patient with SCoVaG was an 82y old female who developed GBS two weeks after the first dose of an mRNA-based SARS-CoV-2 vaccine (6). Since she did not undergo nerve conduction studies (NCSs), no GBS subtype could be defined (6). She did not require mechanical ventilation. She benefitted from IVIGs, but only partial recovery was achieved (6).

Seven further patients with SCoVaG after administering a vector-based SARS-CoV-2 vaccine were reported from India (Table 1). All these patients had cranial nerve involvement (7). The first patient was a 43y old female who developed back pain 10d after the first dose, which progressed to facial diplegia, quadriparesis, and muscular respiratory insufficiency and required mechanical ventilation. The second patient was a 67y old female who developed limb paresthesias 14d after the first dose, which progressed to facial diplegia, abducens palsy, quadriparesis and respiratory insufficiency and required mechanical ventilation. The third patient was a 53y old female who developed lower limb paresthesias and weakness 12d after the first dose, which progressed to facial diplegia, quadruparesis and respiratory insufficiency and required mechanical ventilation. The fourth patient was a 68y old female who developed upper and lower limb paresthesias and muscle weakness that progressed to facial diplegia, dysphagia, and respiratory insufficiency and was treated with mechanical ventilation (Table 1). The fifth patient was a 70y old male who developed facial diplegia, bulbar palsy, and bilateral distal upper and lower limb numbness 11d after the first dose, which progressed to quadriparesis and respiratory insufficiency and required artificial ventilation (7). The sixth patient was a 60y old female who developed facial diplegia, bulbar palsy, ophthalmoplegia, quadriparesis, and distal upper and lower limb numbness 12d after the first dose (7). She did not develop affection of the respiratory muscles. The seventh patient was a 69y old female who developed facial diplegia, bulbar palsy, and quadriparesis with upper and lower limb numbness 13d after the first dose. She developed respiratory muscle involvement and required artificial ventilation (7).

The 60y old female reported by Marquez-Loza et al. (8) developed back and leg pain followed by headache, nausea, vomiting, double vision, facial diplegia, and paraparesis of lower legs. She benefitted from IVIG and recovered partially (8). NCSs revealed the absence of late responses, being interpreted as early signs of GBS.

The patient reported by Narasimhalu et al. (9) is a 32y old female who developed numbness, swelling, and pain over the left face and neck. She was diagnosed with sensory radiculitis C2/3 and treated with pregabalin (9). Her MRI revealed perineural sheath enhancement V/3. No cerebrospinal fluid (CSF) investigations or NCSs were carried out (9).

Concerning the case reported by Aomar-Millan et al. (10), it remains unclear if acute, motor, and sensory axonal neuropathy (AMSAN) was due to SARS-CoV-2 infection or vaccination that he received before the onset of GBS. The second patient reported by these authors experienced GBS most likely due to SARS-CoV-2 infection and not due to vaccination he received before the infection (10).

The 37y old male reported by Patel et al. (11) presented with progressive quadriparesis and distal sensory disturbances without involving respiratory muscles. He responded only partially to IVIGs (11).

The patient with SCoVaG, as reported by Sadoff et al. (12), was not included in the present review as no details were provided.

According to the Medicines and Healthcare products Regulatory Agency (MHRA), six patients with SCoVaG were further registered, but no details about these patients were reported and hence not included in this review (13).

The Vaccine Adverse Event Reporting System (VAERS) does not list GBS as an adverse reaction to SARS-CoV-2 vaccination (14). However, in the 13^th^ July 2021 update from FDA News Release, it was mentioned that 100 patients developed preliminary SCoVaG after receiving the Johnson & Johnson vaccine (15). In 95 of these patients, GBS was severe, requiring hospitalization. Furthermore, the European Medicines Agency’s (EMA) COVID-19 vaccine safety update indicates that as of 27^th^ June 2021, 227 patients with SCoVaG after receiving the AZV have been reported (16). However, these cases were collected by passive surveillance and were not revised cases. According to the WHO Global Advisory Committee on Vaccine Safety (GACVS) COVID-19 subcommittee on the reports of GBS following adenovirus vector COVID-19 vaccines, only rare cases of SCoVaG have been reported (17). The committee indicates that more rigorous studies using alternative data sources and robust study designs and comparison of vaccinated and unvaccinated populations are warranted (17).

## DISCUSSION

This narrative review shows that since the introduction of the SARS-CoV-2 vaccination in December 2020, at least 19 patients have been reported to experience SCoVaG time-linked to the first dose of a SARS-CoV-2 vaccination. Additionally, more than 300 SCoVaG patients were reported by the FDA and the EMA. In the majority of the 19 cases, SCoVaG developed after the application of a vector-based SARS-CoV-2 vaccine. The latency between vaccination and onset of SCoVaG was highly variable. The severity of the complications ranged from mild to severe and required mechanical ventilation in six patients. In most cases, the outcome was favourable, but only partial recovery was achieved based on the reporting date.

The presence of a causal relationship between vaccination and the occurrence of SCoVaG remains speculative, but several arguments can be raised in favour and against a causal relationship. Arguments favouring a causal relationship are that SARS-CoV-2 infections are associated with GBS development (1); GBS occurred time-linked to the vaccination; the vaccination stimulates the production of T-cells and antibodies, which could cross-react with the structures of the nerve roots; mRNA-based vaccines require modifications to ensure stability while avoiding pathogen-associated molecular patterns that may trigger an excessive inflammatory response (9); mRNA-based vaccines require utilizing lipid nanoparticle encapsulation to reach the intracellular machinery, which has been implicated in causing anaphylaxis (9); and SCoVaG responds to IVIGs and steroids. Arguments against a causal relationship are that the number of reported patients so far is low, that the latency was fairly long in one patient (39d), that the latency was extremely short in another patient (3h), that temporal association does not imply causality, and that GBS could have developed by chance as well. One would expect to see 900-2200 patients developing GBS within six weeks of receiving 1-dose vaccination (Johnson & Johnson) or 1500-3700 patients developing GBS within ten weeks of 2-dose vaccination (Pfizer and Moderna) (8). It is to be noted that molecular mimicry requires a humoral response that requires 10-14d to develop (9). However, the immune-mediated inflammatory response is another postulated mechanism requiring less development time than molecular mimicry (9).

A limitation of this review is that GBS was not diagnosed based on the validated Brighton criteria, which requires CSF investigations and application of NCSs, in all the patients. For diagnosing GBS based on the Brighton criteria, abnormal NCSs increased CSF protein, and CSF cell counts <50 cells/ml are required. In the study by Allen et al. (1), the limitations are that the outcome of the four presented cases was not described in detail, and all four patients did not undergo NCSs. In the study of Narasimhalu et al. (9) the limitation exists that neither NCSs nor CSF studies had been carried out.

In conclusion, this review illustrates that GBS may develop time-linked to the first dose of a SARS-CoV-2 vaccination. Whether there is a causal relationship between vaccination and GBS remains speculative. Still, more arguments in favour than against a causal relationship can be raised, suggesting that GBS can complicate SARS-CoV-2 vaccination in single cases. Despite adequate treatment, some patients may not recover completely. Those involved in the management of SARS-CoV-2 vaccination should remain vigilant for severe side effects in single patients. Early recognition and treatment of GBS may improve the outcome.

## AUTHOR CONTRIBUTIONS

Finsterer J was responsible for the design, literature search, discussion, manuscript first draft and critical comments. Scorza FA and Scorza CA were responsible for the literature search, discussion, manuscript critical comments and final approval.

## Figures and Tables

**Table 1:** Patients developing GBS after SARS-CoV-2 vaccination.

Age	Gender	1./.2 dose	Vaccine type	LVG	Treatment	MV	Outcome	Reference
54	m	first	AZV	12d	St	no	nr	(3)
20	m	first	AZV	21d	St	no	nr	(3)
57	m	first	AZV	11d	IVIG	no	nr	(3)
55	m	first	AZV	22d	none	no	nr	(3)
32	m	first	AZV	8d	IVIG*	no	pr	(4)
69	f	first	AZV	39d	IVIG	no	cr	(submitted)
86	f	first	Pfizer	1d	IVIG	no	pr	(5)
82	f	first	Pfizer	14d	IVIG	no	pr	(6)
43	f	first	AZV	10d	IVIG	MV	nr	(7)
67	f	first	AZV	14d	IVIG	MV	nr	(7)
53	f	first	AZV	12d	nr	MV	nr	(7)
68	f	first	AZV	14d	nr	MV	nr	(7)
70	m	first	AZV	11d	IVIG	MV	pr	(7)
69	f	first	AZV	12d	IVIG,PE	no	pr	(7)
69	f	first	AZV	13d	IVIG	MV	pr	(7)
60	f	first	JJ	17d	IVIG	no	pr	(8)
52	f	first	Pfizer	3h	St,PGB	no	pr	(9)
77	m	first	Pfizer	3d	IVIG,PE	no	nr	(10)
7	m	first	AZV	14d	IVIG	no	pr	(11)

AZV: Astra Zeneca vaccine, cr: complete recovery, f: female, LVG: latency between vaccination date and onset of GBS, m: male, JJ: Johnson & Johnson vaccine, MV: mechanical ventilation, nr: not reported, PGB: pregabalin, pr: partial recovery, PE: plasma exchange, St: steroids, *: the patient received two cycles, plasmapheresis, and is now undergoing immune-adsorption.

## References

[1] Finsterer J, Scorza FA (2021). Guillain-Barre syndrome in 220 patients with COVID-19. Egypt J Neurol Psychiatr Neurosurg.

[2] Finsterer J, Scorza FA (2021). SARS-CoV-2 vaccines are not free of neurological side effects. Acta Neurol Scand.

[3] Allen CM, Ramsamy S, Tarr AW, Tighe PJ, Irving WL, Tanasescu R (2021). Guillain-Barré Syndrome Variant Occurring after SARS-CoV-2 Vaccination. Ann Neurol.

[4] Finsterer J (2021). Exacerbating Guillain-Barré Syndrome Eight Days after Vector-Based COVID-19 Vaccination. Case Rep Infect Dis.

[5] Ogbebor O, Seth H, Min Z, Bhanot N (2021). Guillain-Barré syndrome following the first dose of SARS-CoV-2 vaccine: A temporal occurrence, not a causal association. IDCases.

[6] Waheed S, Bayas A, Hindi F, Rizvi Z, Espinosa PS (2021). Neurological Complications of COVID-19: Guillain-Barre Syndrome Following Pfizer COVID-19 Vaccine. Cureus.

[7] Maramattom BV, Krishnan P, Paul R, Padmanabhan S, Cherukudal Vishnu Nampoothiri S, Syed AA (2021). Guillain-Barré Syndrome following ChAdOx1-S/nCoV-19 Vaccine. Ann Neurol.

[8] Márquez Loza AM, Holroyd KB, Johnson SA, Pilgrim DM, Amato AA (2021). Guillain- Barré Syndrome in the Placebo and Active Arms of a COVID-19 Vaccine Clinical Trial: Temporal Associations Do Not Imply Causality. Neurology.

[9] Narasimhalu K, Lee WC, Salkade PR, De Silva DA (2021). Trigeminal and cervical radiculitis after tozinameran vaccination against COVID-19. BMJ Case Rep.

[10] Aomar-Millán IF, de Victoria-Carazo JM, Antonio Peregrina-Rivas J, Villegas-Rodríguez I (2021). Covid-19, guillain-barré y vacuna. Una mezcla peligrosa [(Covid-19, Guillain-Barré Syndrome, And The Vaccine: A Dangerous Combination]. Rev Clin Esp.

[11] Patel SU, Khurram R, Lakhani A, Quirk B (2021). Guillain-Barre syndrome following the first dose of the chimpanzee adenovirus-vectored COVID-19 vaccine, ChAdOx1. BMJ Case Rep.

[12] Sadoff J, Gray G, Vandebosch A, Cárdenas V, Shukarev G, Grinsztejn B (2021). Safety and Efficacy of Single-Dose Ad26.COV2.S Vaccine against Covid-19. N Engl J Med.

[13] Medicines and Healthcare products Regulatory Agency COVID-19 vaccine AstraZeneca analysis print. GOV.UK.

[14] VAERS Vaccine adverse event reporting system.

[15] FDA News Release Coronavirus (COVID-19) update. July 13th 2021..

[16] COVID-19 vaccine safety update of the European Medicines Agency https://www.ema.europa.eu/en/documents/covid-19-vaccine-safety-update/covid-19-vaccine-safety-update-vaxzevria-previously-covid-19-vaccine-astrazeneca-14-july-2021_en.pdf.

[17] World Health Organization Statement of the WHO Global Advisory Committee on Vaccine Safety (GACVS) COVID-19 subcommittee on reports of Guillain-Barré Syndrome (GBS) following adenovirus vector COVID-19 vaccines.

